# A Corner-Analysis-Based Method for Dimensional Inspection and Defect Detection of Glass Chamfers

**DOI:** 10.3390/s26154764

**Published:** 2026-07-27

**Authors:** Jie Jiang, Tian Wang, Yan Zhou, Wan Wang, Congyi Wu, Bing Wei

**Affiliations:** 1 School of Mechanical Engineering, Hubei University of Technology, Wuhan 430068, China; jiejiang_245@163.com (J.J.); 19910049@hbut.edu.cn (B.W.); 2 Shanghai Institute of Space Power-Sources, Shanghai 200245, China; wangtian415@163.com (T.W.); 13817747686@139.com (Y.Z.); 3 School of Economics and Management, Beijing Jiaotong University, Beijing 100044, China; 4 School of Mechanical Science and Engineering, Huazhong University of Science and Technology, Wuhan 430074, China; wucongyi@hust.edu.cn; 5 Guangdong HUST Industrial Technology Research Institute, Dongguan 523808, China; 6 Guangdong Intelligent Robotics Institute, Dongguan 523808, China

**Keywords:** glass chamfer, machine vision, corner detection, image processing, dimensional measurement, defect detection

## Abstract

Edge chamfering is a critical process in glass manufacturing because it suppresses crack propagation and improves both edge strength and operational safety. As such, it has become an essential step in modern glass production. However, in current automated production lines, chamfer quality inspection still relies heavily on manual visual inspection and contact-based measuring instruments, which are limited by low efficiency, strong subjectivity, and poor compatibility with production-line takt time. To overcome these limitations, this paper proposes a vision-based inspection method for transparent glass chamfers using corner analysis, and a dedicated glass chamfer inspection system is developed for experimental validation. First, machine-vision image processing is applied to enhance chamfer-region features and improve detection robustness. Candidate endpoint generation is then combined with the construction of an endpoint-screening coordinate system to achieve stable localization of chamfer endpoints. In addition, quadrant-based spatial constraints are introduced to further enhance endpoint localization stability, enabling automatic measurement of key geometric parameters, including chamfer length, width, and height. On this basis, multiple defect-discrimination criteria are established by integrating geometric tolerance violations with spatial abnormalities in endpoint distribution, thereby enabling automatic identification of chamfer defects. Experimental results show that the proposed method achieves a mean absolute measurement error of 0.087 mm and an average inspection time of 0.505 s per workpiece. The method can also effectively identify chamfer defects, demonstrating its suitability for online inspection while maintaining both dimensional measurement accuracy and defect detection capability.

## 1. Introduction

Edge chamfering is a key process in glass manufacturing, as it helps suppress crack initiation and improve service safety [1]. A properly designed chamfer geometry can effectively reduce stress concentration at the glass edge, inhibit crack propagation toward the glass surface, decrease the risk of mechanical damage, and enhance the bending strength of the chamfered edge [2]. Previous studies have shown that the edge region of glass components is typically one of the most vulnerable areas for crack initiation and fracture. Its mechanical performance is affected not only by the intrinsic brittleness of the material, but also by surface and subsurface defects introduced during edge-processing operations, such as cutting, grinding, and polishing [3,4,5]. For glass subjected to edging and chamfering, the morphology of microcracks, residual stress state, and processing consistency of the edge surface, chamfered surface, and their transition regions can further influence fracture behavior and load-bearing capacity [6,7]. In industrial production, glass chamfers are commonly formed by grinding and polishing with abrasive wheels. However, wheel wear, fluctuations in process parameters, and inconsistencies among different processing devices may all lead to variations in chamfer dimensions, edge quality, and strength performance, thereby affecting subsequent heat treatment, handling, assembly, and final product quality [8,9]. Therefore, after edge chamfering is completed, accurate, stable, and efficient inspection of both geometric dimensions and defect conditions is essential to ensure product safety and manufacturing quality. At present, manual visual inspection combined with contact-based measuring tools remains the primary method for chamfer dimension measurement and defect detection on production lines. However, these methods are often inadequate for identifying small defects and subtle dimensional deviations. Moreover, contact measurement may cause mechanical damage or secondary defects, while point-by-point measurement is time-consuming and difficult to reconcile with production-line takt time requirements. Therefore, a high-precision, high-efficiency, and non-contact method for glass chamfer inspection is urgently needed.

A substantial body of research has focused on machine vision-based inspection and measurement technologies, mainly because they are non-contact, highly automated, fast, and readily integrated into industrial production lines. In the field of industrial surface quality inspection, Chen et al. [10] reviewed defect detection methods for industrial product surfaces and pointed out that both conventional vision-based approaches based on texture, color, and shape features, as well as deep learning-based detection frameworks, have been widely applied to surface quality inspection. Fang et al. [11] systematically summarized the development of automated visual surface defect detection techniques for flat industrial metal materials and classified two-dimensional inspection methods into statistical, spectral, model-based, and machine learning categories. Neogi et al. [12] reviewed visual inspection systems for steel surfaces and concluded that vision-based inspection has become an important technical route for the automated evaluation of steel surface quality. Ren et al. [13] further summarized the current research status and development trends from the broader perspective of machine vision-based defect inspection. Beyond general industrial surfaces, machine vision has also made notable progress in the inspection of transparent components and glass products. Martínez et al. [14] developed an industrial vision system for surface quality inspection of transparent parts, thereby demonstrating the feasibility of machine vision for transparent-part defect detection. Zauner and Schagerl [15] proposed a machine vision scheme for flat-glass product quality inspection and achieved the identification of multiple types of glass defects. Li et al. [16] presented a defect detection and extraction method based on principal component analysis for smartphone cover glass, which was capable of identifying scratches, cracks, deformation, edge chipping, and abnormal corner cutting. Jiang et al. [17] employed a symmetric convolutional neural network for surface defect detection in smartphone back-cover glass. Mao et al. [18] further introduced deep learning into smartphone glass surface defect inspection to improve detection performance in complex scenarios. More recently, Ding et al. [19] developed an automatic optical inspection method for mobile phone flat glass by combining total reflection lighting, defect segmentation, data augmentation, and object detection, showing the potential of deep-learning-assisted methods for glass defect inspection. Li et al. [20] proposed a DY-YOLO model for smartphone cover glass defect detection and demonstrated improved detection accuracy and efficiency under complex backgrounds and multi-scale defect conditions. In addition, recent reviews and studies on industrial surface defect detection have shown that deep-learning-based methods have achieved substantial progress, but they still face challenges related to small defects, defect-scale variation, data scarcity, annotation cost, and the balance between accuracy and real-time performance [21,22]. At the same time, machine vision has been widely used in dimensional measurement and manufacturing quality control. Gadelmawla [23] proposed a computer vision algorithm for the measurement and inspection of external screw threads. Moru and Borro [24] developed a machine vision inspection algorithm for gear quality control and achieved subpixel-level measurement accuracy. Taatali et al. [25] investigated on-machine vision-based dimensional inspection for CNC-machined parts. Li et al. [26] proposed a deep learning-based method for online visual dimensional measurement of workpieces. Hashmi et al. [27] provided a review of computer vision-based surface feature measurement. In addition, machine vision has shown strong applicability in other scenarios as well. For example, Sánchez-Jiménez et al. [28] developed a computer vision application for measuring skin wound dimensions, whereas Silva and Paladini [29] proposed an intelligent machine vision system for the automatic verification of multiple components and threaded parts on automotive assembly lines. Overall, recent studies indicate that machine vision and deep-learning-based inspection methods have been widely applied in industrial surface quality evaluation, transparent-part and glass inspection, precision measurement, and intelligent manufacturing. However, most existing studies still focus on either general surface defect detection or dimensional measurement as separate tasks. For transparent glass chamfers, several technical challenges remain insufficiently addressed. First, weak contrast and light transmission characteristics make chamfer endpoints and edge defects difficult to extract reliably. Second, conventional edge- or contour-based methods are sensitive to local edge discontinuities, contamination, and background interference. Third, deep-learning-based methods usually require a large number of well-annotated defect samples, which are difficult to obtain in production-line scenarios. Therefore, targeted methods that can simultaneously achieve chamfer dimensional measurement and defect assessment under weak-contrast imaging conditions remain limited.

On this basis, this study proposes a corner-analysis-based vision inspection method for transparent glass chamfers and develops a corresponding experimental system for validation. First, the acquired images are preprocessed to enhance weak chamfer-region features and improve detection robustness. Candidate corners are then generated and further screened through contour fitting, an offset coordinate system, and quadrant-based geometric constraints, enabling stable localization of chamfer endpoints and automatic measurement of chamfer dimensional parameters. In this study, detection robustness refers to the ability of the proposed inspection method to maintain reliable chamfer endpoint localization and defect discrimination under weak-contrast imaging conditions and typical production-line disturbances, such as uneven illumination, local noise, glass debris, and slight variations in workpiece position. Building on the endpoint localization results, typical chamfer defects are identified according to dimensional tolerance violations and abnormal spatial distributions of endpoint features. The proposed method provides a non-contact, interpretable, and production-oriented solution for online glass chamfer quality inspection. The main contributions of this study are summarized as follows: (1) A corner-analysis-based endpoint localization method is proposed for transparent glass chamfers by integrating candidate corner detection, contour fitting, an offset coordinate system, and quadrant constraints. (2) A unified vision-based inspection framework is developed for both chamfer dimensional measurement and defect detection, enabling the automatic evaluation of chamfer length, width, height, and typical abnormal conditions. (3) The proposed rule-based method does not require large-scale labeled defect samples and provides an interpretable and efficient solution for online glass chamfer inspection in industrial production.

## 2. Principles and Methods

### 2.1. Glass Chamfer In-Line Vision-Based Inspection Device Construction

**Figure 1:**
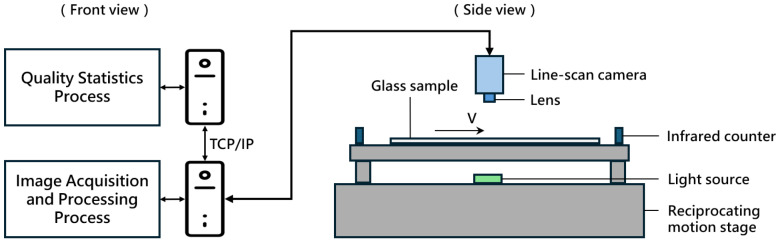
Structure of the Glass Chamfer Measurement System.

To meet the requirements of real-time measurement and defect inspection for glass chamfers, a machine vision-based chamfer inspection system was developed in this study. The system mainly consists of an industrial camera and lens assembly, a light source and its controller, mounting brackets and positioning fixtures, a reciprocating motion mechanism for the glass workpiece under inspection, and an industrial computer equipped with image acquisition and processing software. The overall configuration of the system is shown in Figure 1.

**Figure 2:**
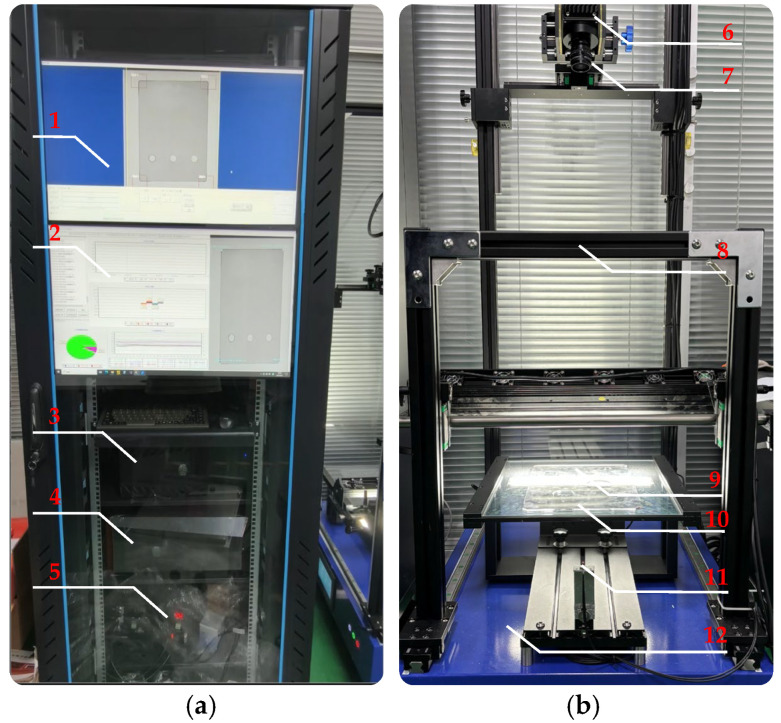
Glass Chamfer Measurement System: (**a**) Software platform; (**b**) Hardware platform. The numbered components are as follows: (1) management software, (2) data statistics and analysis software, (3, 4) computing-platform hosts, (5) light-source controller, (6) industrial camera, (7) industrial lens, (8) support structure, (9) white backlight source, (10) glass sample, (11) infrared counter, and (12) reciprocating motion platform.

A photograph of the overall inspection system is shown in Figure 2. The system acquires images of glass chamfers through the coordinated use of the industrial camera, lens, and backlight illumination, while synchronized inspection of the workpiece is achieved through the triggering unit and motion mechanism. Image processing, result determination, and statistical data analysis are then performed by the industrial computer.

The system utilizes a Teledyne DALSA 8K line-scan industrial camera (Teledyne DALSA, Waterloo, ON, Canada), paired with a Camera Link frame grabber for image acquisition, and camera control and data capture are implemented via the Teledyne DALSA Sapera LT SDK (Teledyne DALSA, Waterloo, ON, Canada). The lens selected is a Schneider-Kreuznach Apo-Componon 60 mm industrial lens (Jos. Schneider Optische Werke GmbH, Bad Kreuznach, Germany) with a maximum aperture of f/4.0. To achieve synchronized image acquisition with the line-scan camera, the OMRON E3Z-T61 (OMRON Corporation, Kyoto, Japan) infrared counter is configured as the triggering device. The illumination setup employs a high-brightness LED bar backlight to enhance the weak-contrast edges and endpoint features of the transparent glass chamfer.

**Figure 3:**
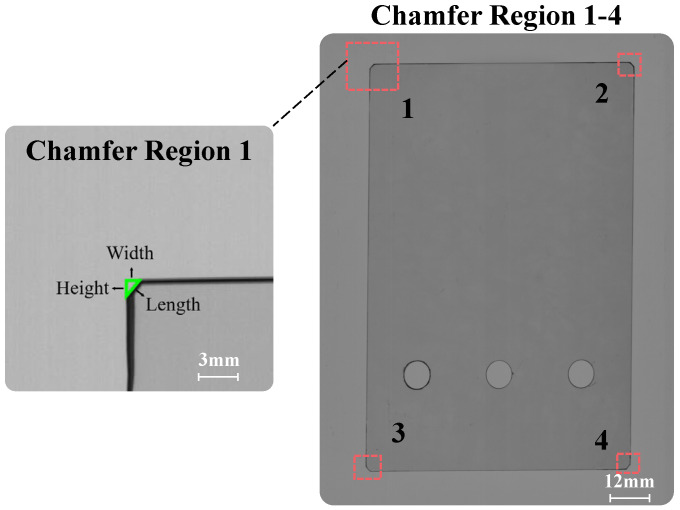
Chamfer geometric information diagram.

The inspection target in this study is the edge chamfer of a transparent rectangular glass workpiece, as shown in Figure 3. Although the chamfered surface has been mechanically formed, its quality still requires online inspection to ensure assembly accuracy and aesthetic standards. The inspection system must accomplish two core tasks: first, to accurately measure key dimensional parameters of the chamfer, including length, width, and height, and evaluate their compliance with design tolerances; second, to detect abnormal features in the chamfer endpoints and edge regions during dimensional measurement, enabling identification of typical defects. To ensure consistency in the definition of geometric parameters and measurement locations, a unified specification for chamfer parameter measurement positions is adopted, as illustrated in Figure 3. All subsequent experiments and result analyses are based on this definition.

### 2.2. Calibration of a Line-Scan Camera

#### 2.2.1. Establishment of the Calibration Model

**Figure 4:**
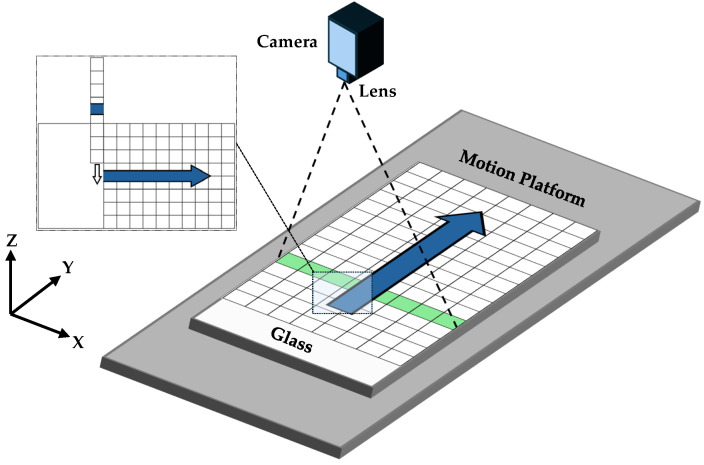
Schematic diagram of the two-dimensional imaging principle of a line-scan camera.

To accurately measure geometric parameters such as chamfer length, width, and height, it is necessary to establish a mapping between image pixel coordinates and actual physical coordinates. In a line-scan imaging system, the dimensions of target features are initially represented in pixel units, whereas subsequent measurements require their corresponding physical dimensions. Therefore, pixel scale factors are used to convert image coordinates into physical coordinates. Unlike an area-scan camera, which captures a two-dimensional image in a single exposure, a line-scan camera acquires only one row of pixels per exposure, and the two-dimensional image of the target is formed by sequentially stitching rows as the workpiece moves relative to the camera. Consequently, line-scan images exhibit different imaging mechanisms along the two directions [30]. Specifically, the lateral x-direction corresponds to the spatial sampling direction of a single line-scan exposure, while the longitudinal y-direction corresponds to the scanning direction generated by the combined effect of workpiece motion and continuous camera sampling. The imaging principle is illustrated in Figure 4.

**Figure 5:**
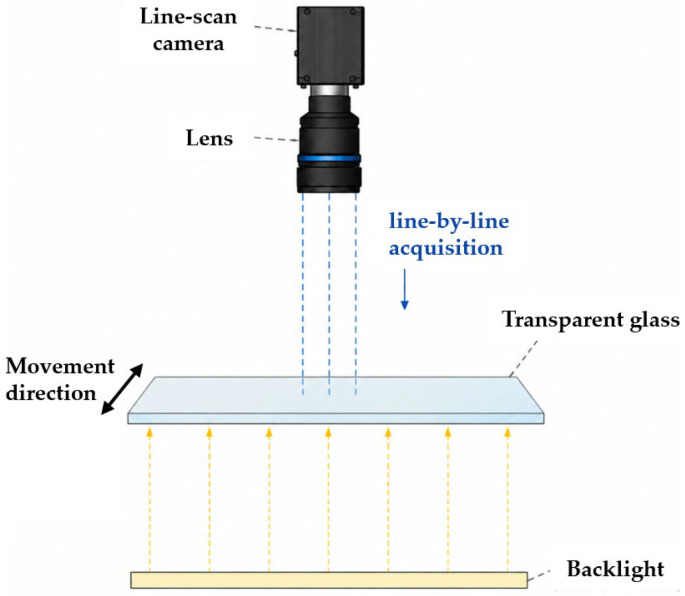
Schematic diagram of backlight illumination system.

To ensure a clear and stable edge profile of the transparent glass, a backlight illumination configuration was adopted during image acquisition. As shown in Figure 5, the strip light source was arranged beneath the glass, and the emitted light passed through the transparent glass before being captured by the line-scan camera through the lens. In this configuration, the glass edge produces a distinct intensity transition in the acquired image, which is beneficial for subsequent edge detection and line extraction. Meanwhile, the backlight arrangement can reduce the influence of surface reflection and uneven ambient illumination, thereby improving the stability of the extracted edge position. During image acquisition, the glass moved along the scanning direction relative to the camera, and the line-scan camera continuously acquired image lines to form a two-dimensional image. This illumination and acquisition setup provides stable image contrast for the subsequent pixel-scale calibration and dimensional measurement.

Under conditions of fixed working distance, stationary camera installation, and a relatively small measurement area, the region can be approximated as a locally linear mapping area. Let H denote the actual length of the target, h the corresponding pixel length in the image, D the distance from the camera to the object, and f the lens focal length. The actual target size and its image pixel size then satisfy the approximate proportional relationship H/h≈D/f. Once the system is installed, D and f remain constant, so D/f can be treated as a constant. Accordingly, the actual size of the target can be expressed as H=Kh, where K is the pixel scale factor in units of mm/pixel. In this study, the unified scale factor K is decomposed into separate scale factors for the x- and y-directions, denoted as $k_{x}$ and $k_{y}$, respectively.

Let (u,v) denote the pixel coordinates of any point in the line-scan image, and (X,Y) the corresponding physical coordinates. Under conditions of negligible perspective distortion and fixed working distance, the measurement area can be approximated as a locally linear mapping region [30], giving:

(1) $$X=k_{x}u+b_{x}$$

(2) $$Y=k_{y}v+b_{y}$$

where $k_{x}$ and $k_{y}$ denote the pixel scale factors in the x- and y-directions, respectively, with units of mm/pixel, and $b_{x}$ and $b_{y}$ represent the coordinate-origin offsets. Since this study focuses on relative dimensional measurements, such as length and width, the origin offsets do not affect the calculation of these dimensions. Therefore, only the scale factors $k_{x}$ and $k_{y}$ need to be determined in the subsequent analysis.

In this expression, $k_{x}$ and $k_{y}$ represent the pixel scale factors in the x- and y-directions, respectively, in units of mm/pixel, while $b_{x}$ and $b_{y}$ denote the coordinate offsets. Since this study focuses on relative dimension measurements such as length and width, the coordinate offsets have no effect on the calculation of lengths. Therefore, only the scale factors $k_{x}$ and $k_{y}$ are used in subsequent calculations, with $b_{x}$ and $b_{y}$ assumed to be zero.

When the pixel coordinates of two points in the image are $\left(u_{1},v_{1}\right)$ and $\left(u_{2},v_{2}\right)$ their corresponding lateral and longitudinal distances in physical space can be expressed as:

(3) $$\Delta X=k_{x}\left|u_{2}-u_{1}\right|$$

(4) $$\Delta Y=k_{y}\left|v_{2}-v_{1}\right|$$

The corresponding Euclidean distance in physical space can be expressed as:

(5) $$L=\sqrt{{\left(\Delta X\right)}^{2}+{\left(\Delta Y\right)}^{2}}=\sqrt{{\left[k_{x}\left(u_{2}-u_{1}\right)\right]}^{2}+{\left[k_{y}\left(v_{2}-v_{1}\right)\right]}^{2}}$$

It can thus be seen that, once the scale factors $k_{x}$ and $k_{y}$ are obtained, the image-based measurement results can be converted into actual physical dimensions.

#### 2.2.2. Scale Calibration in the *x*-Direction

Since the x-direction corresponds to the spatial sampling direction of a single line acquired by the line-scan camera, its scale is mainly determined by the optical magnification of the imaging system and the installation conditions. When the camera position and working distance are fixed, the pixel scale factor in the x-direction can be directly calibrated using a standard reference block. Let $L_{x0}$ denote the actual length of the standard block in the x-direction, and $N_{x}$ the corresponding pixel length in the acquired image. The pixel scale factor in the x-direction obtained from a single measurement can then be expressed as:

(6) $$k_{x}=\frac{L_{x0}}{N_{x}}$$

**Figure 6:**
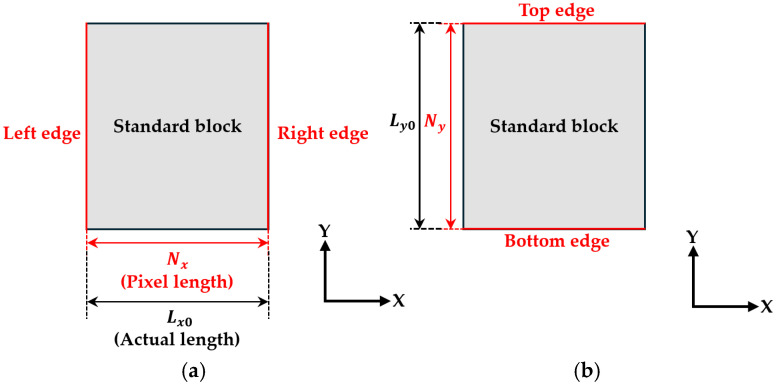
Schematic diagram of pixel length extraction in the *x* and *y*-direction: (**a**) x-direction pixel-length extraction; (**b**) y-direction pixel-length extraction.

Figure 6 shows the extraction process of $N_{x}$, where the pixel length is determined by the distance between the left and right edges of the standard block. Combined with the known actual length $L_{x0}$, the x-direction pixel scale factor is calculated using Equation (6).

To reduce the influence of edge-extraction errors and random noise, repeated measurements are performed so as to suppress incidental errors. Suppose that m repeated experiments are conducted, and the pixel length measured in the i-th experiment is denoted by $N_{x,i}$. Then, the corresponding scale factor obtained in the i-th experiment can be expressed as follows:

(7) $$k_{x,i}=\frac{L_{x0}}{N_{x,i}},i=1,2,\cdots ,m$$

The average value is taken as the final calibration result in the x-direction:

(8) $$\bar{k_{x}}=\frac{1}{m}\sum_{i=1}^{m}k_{x,i}$$

Meanwhile, to evaluate the dispersion of the calibration results, the standard deviation $\sigma_{k_{x}}$ and the relative dispersion coefficient $\eta_{k_{x}}$ are introduced, which are defined as follows:

(9) $$\sigma_{k_{x}}=\sqrt{\frac{1}{m-1}{\sum_{i=1}^{m}\left(k_{x,i}-\bar{k_{x}}\right)}^{2}}$$

(10) $$\eta_{k_{x}}=\frac{\sigma_{k_{x}}}{\bar{k_{x}}}\times 100\%$$

**Figure 7:**
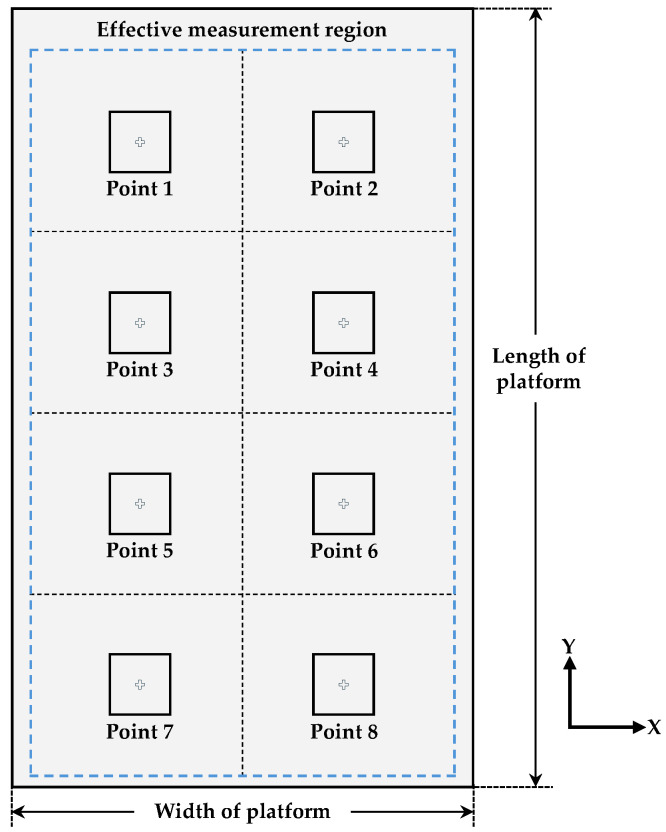
Distribution of calibration positions within the effective measurement region.

In the experiment, a standard block with cross-sectional dimensions of 210 mm×300 mm was selected as the calibration target and placed at the system’s normal inspection position. Images were acquired under identical illumination conditions and working distances. Edge detection and line extraction methods were used to determine the positions of the two edges of the standard block, from which the pixel length $N_{x}$ in the image was obtained, and the pixel scale factor in the x-direction was calculated accordingly. To evaluate the consistency of the x-direction scale within the effective measurement area, the standard block was positioned at different locations on the platform for repeated calibration. The distribution of these calibration positions is shown in Figure 7.

Each measurement point was numbered according to its spatial location, and the calibration results are presented in Table 1.

As shown in Table 1, the variation of $k_{x}$ across different positions is minimal. Therefore, a unified x-direction scale factor can be approximately used for subsequent measurements, with the final average value set as $\bar{k_{x}}=0.09546\;$ mm/pixel.

#### 2.2.3. Scale Calibration in the *y*-Direction

Unlike the x-direction, which is determined by the lateral imaging of the line-scan camera, the y-direction corresponds to the scanning direction formed by the relative motion of the workpiece with respect to the camera, driven by the motion stage. Its scale is mainly determined by the stage displacement and the line-triggering mode of the camera. Considering that the reciprocating motion stage exhibits speed variation near the reversal positions, image acquisition and dimensional measurement are both performed within the stable constant-speed interval of stage motion, while avoiding the non-steady phases near reversal, so as to ensure the accuracy and consistency of dimensional conversion in this direction. During image acquisition, each acquisition sequence is initiated by an external trigger signal, whereas the synchronization signal for each scan line is provided by the input from the shaft encoder. Therefore, the pixel scale factor in the y-direction is not directly determined by a fixed free-running line frequency inside the camera, but rather jointly determined by the encoder pulse equivalent and the trigger division ratio.

Let the encoder calibration value be P pulses/mm. Then, the actual displacement corresponding to one pulse is $d_{e}=1/P$. In the present system, the encoder calibration value is P=50 pulses/mm; therefore, the actual displacement corresponding to a single pulse is $d_{e}=1/50=0.02$ mm/pulse. If the system is configured such that one image line is acquired for every accumulated n encoder pulses, then the actual length corresponding to one pixel in the y-direction, namely the longitudinal pixel scale factor, can be expressed as follows:

(11) $$k_{y}=nd_{e}$$

Substituting $d_{e}=0.02$ mm/pulse into the above equation yields $k_{y}=0.02n$. In the proposed inspection system, the acquisition is configured such that one image line is triggered for every four encoder pulses, i.e., n=4. Therefore, according to the theoretical trigger parameters of the system, the theoretical pixel scale factor in the y-direction is $k_{y,th}=4\times 0.02=0.08$ mm/pixel. This result indicates that, under ideal conditions, the system acquires one line of image data for every 0.08 mm  of stage motion. Accordingly, each additional pixel in the longitudinal direction of the image corresponds to an actual workpiece displacement of approximately 0.08 mm.

On the other hand, if the motion velocity of the stage during the constant-speed interval is v, then the corresponding equivalent line frequency of the system can be expressed as follows:

(12) $$f_{eq}=\frac{v}{k_{y}}$$

Within the effective inspection range, the proposed detection system maintained a constant speed of v=50 mm/s. Substituting the theoretical scale factor $k_{y,th}=0.08\;$ mm/pixel into the above equation yields the corresponding equivalent line frequency of the system as $f_{eq}=50/0.08=625\;\mathrm{Hz}$. Here, $f_{eq}$ denotes the equivalent line frequency converted from the platform speed and the pulse-trigger relationship; essentially, it reflects the average number of sampled lines per unit time rather than the fixed internal line frequency set by the camera in free-run mode. To further obtain a y-direction parameter that better reflects the characteristics of the actual measurement system, repeated calibration experiments of the longitudinal pixel scale factor were conducted at different positions using a standard reference block. The calibration results are presented in Table 2.

The experimental results show that the actual effective longitudinal pixel scale factor of the system is $\bar{k_{y,exp}}=0.08283$ mm/pixel. Comparing the experimental value with the theoretical value, the absolute difference between them is $\Delta k_{y}=|0.08283-0.08|=0.00283$ mm/pixel, and the relative deviation is given by:

(13) $$\delta_{y}=\frac{\left|\bar{k_{y,\exp}}-k_{y,th}\right|}{k_{y,th}}\times 100\%=3.54\%$$

According to the relative deviation, a certain discrepancy exists between the theoretical value and the experimental value. This discrepancy is mainly caused by the combined effects of actual stage motion error, encoder conversion error, and experimental measurement error. Considering that the experimentally calibrated value can more faithfully reflect the longitudinal scale characteristics of the entire inspection system under actual operating conditions, the experimental calibration value is adopted in the subsequent dimensional conversion as the final pixel scale factor in the y-direction, namely $k_{y}=0.08283$ mm/pixel. In summary, the pixel scale factor in the y-direction is first theoretically derived from the encoder pulse equivalent and trigger settings, and is then experimentally calibrated using a standard target to obtain the actual effective value of the system. In subsequent measurements, 0.08283 mm/pixel is used as the dimensional conversion coefficient in the y-direction.

#### 2.2.4. Calibration Error Analysis and Validation of Results

To further evaluate the effectiveness, repeatability, and stability of the calibrated pixel scale factors, validation experiments were carried out after completing the scale calibration in both the x- and y-directions. Specifically, the standard block was repositioned at different locations within the measurement field, and its edge dimensions were measured repeatedly under the same imaging conditions. The previously calibrated scale factors were then applied to convert the measured pixel values back into the corresponding physical dimensions of the standard block. In total, eight independent validation experiments were performed to assess the reliability of the calibration results. The detailed validation data and calculated dimensional errors are presented in Table 3 and Table 4.

As shown in Table 3, in the x-direction, the mean back-calculated dimension of the standard block is 209.888 mm, with a mean error of 0.172 mm and a maximum error of 0.351 mm.

As shown in Table 4, in the y-direction, the mean back-calculated dimension is 299.921 mm, with a mean error of 0.239 mm and a maximum error of 0.418 mm. These results indicate that the dimensions obtained using the calibrated pixel scale factors are generally close to the true dimensions of the standard block, demonstrating that the established scale conversion relationship has good stability and consistency within the effective measurement region.

The above validation results are based on the relatively large dimensional range of the standard block, and essentially reflect the accumulated conversion error of the pixel scale factor over a large measurement span. In contrast, the subsequent chamfer measurements in this study focus on local regions near the workpiece edges, where the corresponding pixel spans are significantly smaller than the full size of the standard block. Under otherwise identical conditions, the error propagated from the scale factor deviations into local chamfer measurements is generally smaller than the back-calculation error observed during calibration validation. Furthermore, the calibration results presented here were obtained with fixed camera installation, working distance, and workpiece motion conditions. When the camera height, workpiece surface position, or relative installation configuration changes significantly, the original pixel scale factors may no longer be valid, and recalibration is required to ensure the accuracy of subsequent dimensional measurements.

### 2.3. Image Pre-Processing

#### 2.3.1. Image Correction

In real-world production-line settings, disturbances such as dust, debris, and localized local illumination contamination are unavoidable. Combined with non-uniform illumination and camera response non-uniformity, these factors cause the captured raw images to exhibit pronounced spatial intensity inhomogeneity, along with sporadic bright and dark artifacts. Such non-uniformity can induce false defects, where background regions are misclassified as foreground in some areas, while in other areas the grayscale contrast at the chamfer edge is reduced. Moreover, corner detection algorithms typically depend on abrupt grayscale changes to identify corner points. Therefore, illumination correction is necessary to ensure that grayscale variations more faithfully represent the true edge characteristics of the object and to mitigate misdetections caused by uneven lighting or artifacts. Accordingly, flat-field correction is applied to the acquired raw images in this study based on the commonly used flat-field normalization principle, in which the raw image is normalized using a uniformly illuminated reference image [31]. When the exposure time and illumination parameters are kept constant, the imaging system can be approximated as follows:

(14) $$I_{raw}\left(x,y\right)=G\left(x,y\right)I_{obj}\left(x,y\right)+n\left(x,y\right)$$

Here, $I_{raw}\left(x,y\right)$ denotes the raw inspection image, $I_{obj}\left(x,y\right)$ represents the ideal intensity distribution of the object, G(x,y) is a spatially slowly varying gain field jointly induced by illumination non-uniformity, the lens transfer characteristics, and the camera response, and n(x,y) is the noise term. Under identical imaging conditions, an image of a uniform white reference target is captured as the flat-field image $I_{white}$. In this case, $I_{obj}\left(x,y\right)\approx I_{0}$, where $I_{0}$ is a constant, and thus $I_{white}\approx {G\left(x,y\right)I}_{0}$. Accordingly, the raw inspection image $I_{raw}$ is normalized to obtain the corrected image as follows:

(15) $$I_{corr}\left(x,y\right)=\frac{I_{raw}\left(x,y\right)}{I_{white}\left(x,y\right)}\cdot \bar{I_{white}}$$

**Figure 8:**
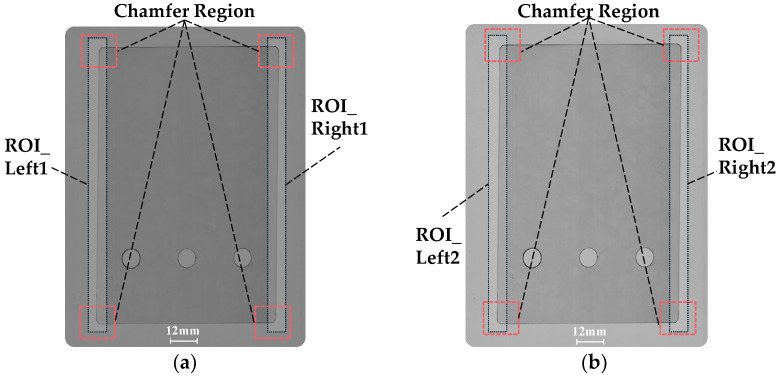

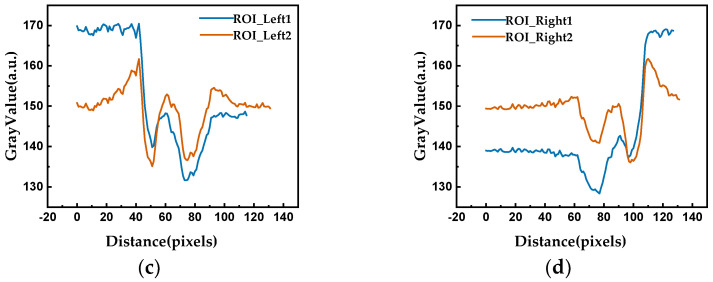
Flat-field correction comparison: (**a**) Original image before flat-field correction with ROI (region of interest); (**b**) Image after flat-field correction with ROI; (**c**) Gray value profiles of ROI_Left; (**d**) Gray value profiles of ROI_Right.

Here, $I_{white}$ denotes the global mean grayscale intensity of the flat-field image and is used to maintain a consistent overall brightness level. The comparison before and after correction is shown in Figure 8.

This correction procedure effectively compensates for slowly varying illumination non-uniformity and vignetting effects, suppresses artifacts induced by an inhomogeneous illumination field, and yields more consistent contrast in the chamfer region, thereby providing a more stable input for subsequent threshold-based segmentation and corner-feature extraction.

#### 2.3.2. Region-of-Interest Extraction

During online inspection of transparent glass chamfers, a prior-knowledge-based ROI extraction strategy is employed to improve image-processing efficiency and reduce the influence of background interference on endpoint localization and dimensional measurement. When inspection begins, the system first acquires a test image, and based on the nominal dimensions and allowable tolerances of the chamfers in the current batch, the ROI of each chamfer region is manually predefined. Because the orientation of glass workpieces on the production line is relatively stable and the loading position and angle vary minimally, the predefined ROIs can consistently cover the corresponding chamfer regions during continuous inspection. This approach effectively limits the area for subsequent image processing, thereby enhancing detection efficiency and system stability.

#### 2.3.3. Image Filtering

**Figure 9:**
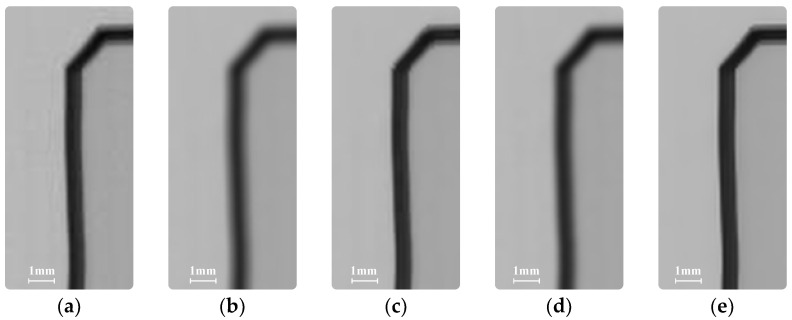
Comparison of chamfer-edge images under different filtering methods: (**a**) original image, (**b**) mean filtering, (**c**) median filtering, (**d**) Gaussian filtering, and (**e**) bilateral filtering.

The filtering methods considered for comparison include mean filtering, median filtering, Gaussian filtering, and bilateral filtering. As shown in Figure 9, in regions where the chamfer edge exhibits low contrast and is contaminated by isolated noise points, mean and Gaussian filtering weaken the grayscale transitions at the edge to varying degrees, resulting in a wider boundary transition zone. Although median filtering can suppress isolated noise points, it may introduce local shape deviations near weak boundaries. In contrast, by jointly accounting for spatial distance and intensity similarity, bilateral filtering can reduce noise while better preserving the Chamfer edge structure [32], thereby producing clearer details in the vicinity of the endpoints. Therefore, bilateral filtering is selected as the smoothing method for subsequent processing in this study.

To provide a more objective justification for the selection of the filtering method, quantitative evaluation was further conducted using peak signal-to-noise ratio (PSNR), structural similarity index (SSIM) and edge preservation index (EPI). The results are summarized in Table 5.

**Figure 10:**
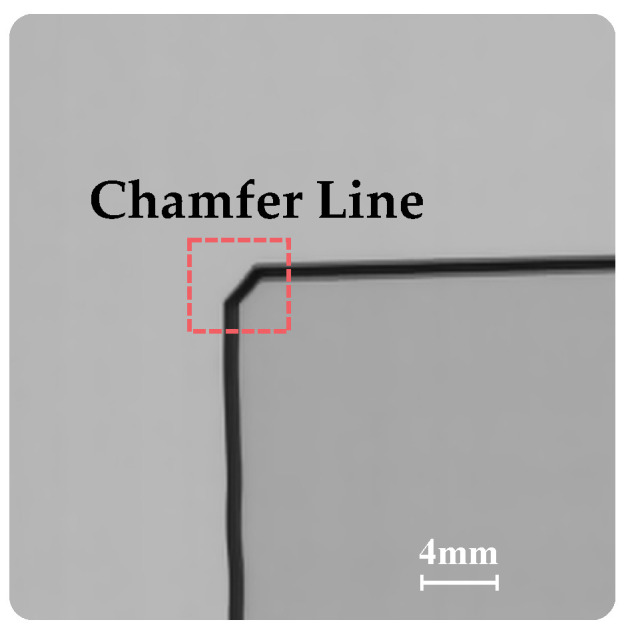
Chamfer feature after bilateral filtering.

As shown in Table 5, bilateral filtering achieved the best overall performance among the four filtering methods, with the highest PSNR, SSIM, and EPI values of 36.8426, 0.9868, and 0.9847, respectively. This indicates that bilateral filtering not only better preserves the overall image structure but also maintains the chamfer-edge information more effectively. Median filtering also produced relatively high SSIM and EPI values, but its PSNR was lower than that of bilateral filtering, suggesting a weaker overall grayscale restoration capability. Mean and Gaussian filtering showed lower EPI values, indicating that they tended to weaken the chamfer-edge transition during noise suppression. Therefore, bilateral filtering was selected as the smoothing method for subsequent contour fitting and endpoint localization. As shown in Figure 10, after bilateral filtering, random background noise and localized speckle-like disturbances are effectively suppressed, while the overall contour continuity and contrast consistency of the chamfer edge are improved. This provides a more stable input for subsequent threshold-based segmentation, contour fitting, and endpoint corner detection, thereby enhancing the robustness of the measurement process and the stability of the results.

### 2.4. Chamfer Endpoint Localization and Dimensional Measurement

#### 2.4.1. Candidate Point Generation Based on Corner Detection

The two endpoints of a glass chamfer line appear in image space as typical geometric discontinuities. On the one hand, along the tangential direction of the chamfer contour, the image intensity exhibits a pronounced transition from the glass body to the background. On the other hand, at the chamfer endpoint, the contour is formed by the intersection of two edges, leading to a distinct change in local curvature. Therefore, under normal processing conditions, chamfer endpoints can be regarded as feature points satisfying the following corner criteria: (1) a significant gradient variation exists along the contour tangential direction; (2) the point corresponds to an extremum of the second derivative along the contour direction; and (3) the curvature in the local neighborhood changes discontinuously. These characteristics are consistent with the classical definition of a corner, in which translating a local window in multiple directions produces substantial intensity variation, thereby providing the theoretical basis for the corner-based chamfer endpoint detection method proposed in this study.

To achieve stable localization of glass chamfer endpoints, a set of candidate corners is first extracted from the preprocessed images to serve as the input for subsequent endpoint screening. In the proposed method, corner detection is primarily used for candidate point generation, with the aim of providing reliable yet not overly dense candidate points in the vicinity of chamfer endpoints while keeping the number of false detections in non-chamfer regions under control. The final endpoints are not determined directly by the corner detector itself, but are selected through a subsequent geometric-constraint-based model. Given the stringent requirements of online production-line inspection in terms of real-time performance and reproducibility, four representative corner detection methods—Harris, Shi–Tomasi, FAST, and ORB—were selected for comparison. To ensure a fair comparison, the same input images and preprocessing pipeline were used for all methods in this section, with only the candidate corner detector being replaced.

The Harris method [33] analyzes grayscale intensity variations induced by translating a local window in different directions, approximates the resulting change as a quadratic form, and thereby derives the structure tensor M, i.e., the gradient covariance matrix, defined as follows:

(16) $$M=\sum_{\left(i,j\right)\in w}w\left(i,j\right)\left[\begin{array}{cc} I_{x}^{2} & I_{x}I_{y}\\ I_{x}I_{y} & I_{y}^{2} \end{array}\right]$$

Here, w(i,j) is a Gaussian weighting function, and $I_{x}$,$I_{y}$ denote the image gradients in the x and y directions, respectively. Based on these gradients, Harris defined the corner response function as follows:

(17) $$R=\det\left(M\right)-k\cdot {\left(trace\left(M\right)\right)}^{2}$$

Let $\lambda_{1}$ and $\lambda_{2}$ denote the eigenvalues of matrix M, where trace(M) = $\lambda_{1}$ + $\lambda_{2}$ and det(M) = $\lambda_{1}$ · $\lambda_{2}$. The parameter k is typically chosen empirically in the range of 0.04–0.06; in our experiments, k = 0.05 is used. The decision rule is as follows: a large R indicates a corner, R < 0 indicates an edge point, and R≈0 corresponds to a flat region. Like Harris, the Shi–Tomasi method is also based on eigenvalue analysis of the local structure tensor, but it uses the minimum eigenvalue as the measure of corner response [34]. FAST performs rapid corner detection by comparing the intensity of a candidate pixel with those of its neighboring pixels, without requiring explicit gradient computation, making it a highly efficient corner detector for real-time applications [35]. ORB further builds on FAST-detected candidate points by incorporating orientation information and feature descriptors to enhance feature representation capability [36].

**Figure 11:**
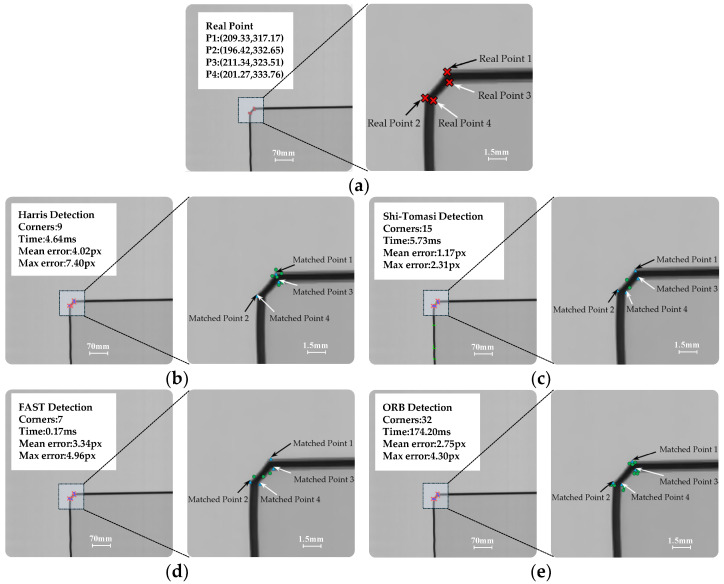
Representative comparison of different corner detectors against ground-truth corners: (**a**) ground-truth annotation, (**b**) Harris, (**c**) Shi-Tomasi, (**d**) FAST, and (**e**) ORB. Green points denote detected candidate corners, blue triangles indicate the detected points closest to the corresponding ground-truth corners, and red crosses mark the ground-truth corners.

The corner coordinates produced by the four methods were all treated as candidate point sets, and endpoint localization and dimensional measurement were performed within the same preprocessing and geometric screening framework to evaluate the effects of different candidate generation strategies on endpoint localization success rate, measurement error, and computational efficiency. Figure 11 shows the candidate point detection results of the different methods on a representative sample. In the figure, the green points denote the potential corners detected by the algorithm, the blue triangles represent the detected candidate points closest to the ground-truth corners, and the red crosses indicate the ground-truth corners. The quantitative statistics for each method on this sample are provided in the upper-left corner of each subfigure, including the number of detected candidate corners (N), runtime (Time), mean error (mean localization error), and max error (maximum localization error).

To further compare the overall performance of different candidate point generation methods across multiple samples, approximately 250 Chamfer images were used for statistical evaluation. Because different corner detectors may fail to generate valid candidate points or complete endpoint matching in a small number of images, the number of valid samples slightly differed among methods. Therefore, the valid sample size for each detector is reported separately in Table 6. It should be noted that Table 6 reports statistical results over multiple samples rather than the detection result of a single image. In the table, N denotes the mean number of candidate corners detected per image; therefore, it can be a decimal value after averaging across multiple samples. Time denotes the average runtime per detection. MLE denotes the mean localization error, which represents the average Euclidean distance between the detected candidate corners and their corresponding manually annotated ground-truth corners. MaxLE denotes the maximum localization error. Recall refers to the average recall rate for successfully covering the ground-truth corners, and PR denotes the Perfect Rate, which represents the proportion of samples in which all ground-truth corners were successfully covered by the detection results. The *p*-value indicates the statistical significance of the MLE difference compared with Shi–Tomasi.

In this study, the ground-truth corners were obtained through manual annotation. Each sample contains four key chamfer corners, corresponding to the two endpoints at both ends of the chamfered region. A ground-truth corner is considered successfully detected if the Euclidean distance between the detected point and the corresponding ground-truth corner is below a predefined threshold of 3 px. If all ground-truth corners in a sample are successfully detected, the sample is counted as a perfect detection.

As shown in Table 6, the four candidate corner detectors differ markedly in the number of candidate points, localization accuracy, and computational efficiency. It should be noted that the comparison in Table 6 focuses on candidate corner generation rather than complete inspection pipeline comparison. Harris produces the fewest candidate corners, with an average of only 5.81 points. Its mean localization error and maximum localization error reach 7.28 px and 11.63 px, respectively, and its recall is only 0.354. These results indicate that Harris lacks sufficient stability in the present inspection scenario and therefore cannot provide reliable candidate points for subsequent endpoint screening. FAST shows a clear advantage in real-time performance, with an average runtime of only 0.202 ms and a recall of 0.925, indicating strong responsiveness to corner features. However, its localization accuracy remains clearly inferior to that of Shi–Tomasi, with a mean localization error of 2.37 px and a maximum localization error of 2.90 px. In addition, FAST generates a relatively small number of candidate points, averaging 6.34, which limits the adequacy of endpoint-neighborhood coverage. ORB yields the largest number of candidate corners, with an average of 34.28 points. Its mean localization error is 1.79 px and its maximum localization error is 2.16 px, indicating generally good localization performance. However, its runtime reaches 224.856 ms, making it difficult to satisfy the real-time requirements of online production-line inspection. Moreover, although ORB detects a relatively large number of candidate points in most samples, the detected points in a few low-contrast or contaminated samples tend to become clustered or spatially shifted. This weakens endpoint coverage and reduces the recall to 0.884. In contrast, Shi–Tomasi delivers the best overall performance in both accuracy and stability. Its mean localization error and maximum localization error are only 1.13 px and 1.42 px, respectively, which are the lowest among the four methods. At the same time, it achieves the highest recall, 0.971, and a Perfect Rate of 88.76%. This indicates that Shi–Tomasi can more consistently cover the true corners in the endpoint neighborhood under different sample conditions. In addition, Shi–Tomasi generates a moderate number of candidate points, averaging 14.67, which is sufficient for subsequent geometry-constrained endpoint screening without introducing a substantial burden from excessive redundant points. Considering detection accuracy, robustness, candidate-point distribution characteristics, and computational efficiency as a whole, Shi–Tomasi was ultimately selected as the candidate corner generator to provide more reliable input for subsequent endpoint localization and dimensional measurement.

#### 2.4.2. Chamfer Endpoint Localization Method Based on Contour Fitting and an Offset Coordinate System

After corner detection, it is still necessary to further screen the candidate point set to identify the target points corresponding to the true chamfer endpoints. To this end, this study proposes a chamfer endpoint localization method based on contour line fitting and the construction of an offset coordinate system. The basic idea is to first establish a local reference coordinate system aligned with the chamfer direction by exploiting the geometric structure of the chamfer contour, and then to stably determine the target endpoints at both ends of the chamfer from the candidate corners by combining quadrant constraints with extremum-based criteria. This method avoids the uncertainty associated with directly identifying endpoints solely on the basis of corner response strength and is therefore better suited to routine quality inspection in industrial production lines.

**Figure 12:**
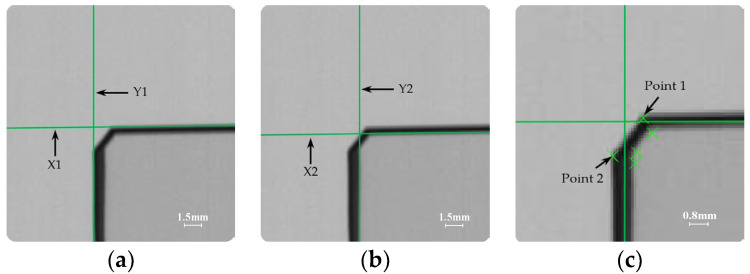
Construction of the screening coordinate system for chamfer endpoint localization: (**a**) Fitted outer boundary line; (**b**) Fitted inner boundary line; (**c**) Corner point screening result.

The chamfer orientation shown in Figure 12 is taken here as the representative case for analysis. To construct the coordinate system for endpoint screening, the linear features of the chamfer contour must first be extracted. Unlike conventional approaches that perform global edge detection prior to line fitting, the present method assumes that the approximate chamfer location is known and that the ROI can be approximately determined from the fixture and installation configuration. Under this condition, only local one-dimensional grayscale profiles are extracted along predefined directions within a small neighborhood. A small number of high-confidence edge points are then identified through gradient-extremum searching, and the corresponding lines are fitted by the least-squares method. This strategy effectively reduces the influence of irrelevant edges, edge discontinuities, and background interference on the fitting results, thereby yielding more stable contour lines under the weak-contrast conditions of transparent glass. Accordingly, the outer chamfer boundary lines $X_{1}$ and $Y_{1}$, together with their corresponding inner boundary lines $X_{2}$ and $Y_{2}$, can be extracted, as shown in Figure 12a,b.

After the inner and outer boundary lines have been obtained, the midpoint positions of the two corresponding edge pairs along their normal directions are used to construct the chamfer endpoint screening coordinate system shown in Figure 12c. Specifically, $X_{1}$ is shifted inward along its normal direction by a distance equal to half of the normal spacing between $X_{1}$ and $X_{2}$, and $Y_{1}$ is shifted inward along its normal direction by half of the normal spacing between $Y_{1}$ and $Y_{2}$. The intersection of these two shifted lines is then defined as the local reference origin, O. On this basis, a local coordinate system consistent with the chamfer geometry is established, in which the X-axis is parallel to the transverse contour of the glass and the Y-axis is perpendicular to it. Using the midpoint between the inner and outer boundaries, rather than a single boundary, as the reference helps reduce the influence of edge extraction fluctuations on spatial endpoint determination and makes the subsequent candidate-point screening more closely aligned with the geometric center zone of the chamfer region. Within the constructed screening coordinate system, the candidate corner points of the chamfer are mapped into different quadrants. For the standard chamfer orientation shown in Figure 12, the two target endpoints are located in the first and third quadrants, respectively; that is, the first endpoint satisfies X>0 and Y>0, while the second satisfies X<0 and Y<0. When the chamfer orientation rotates by 90°, the target endpoints shift correspondingly to the second and fourth quadrants, but their spatial topological relationship remains unchanged. Therefore, quadrant partitioning can serve as the first layer of spatial constraint in endpoint screening, allowing non-target candidate points inconsistent with the chamfer structure to be excluded.

Among the candidate points satisfying the quadrant constraints, the final endpoints are further determined using an extremum-based rule. For the standard chamfer configuration shown in Figure 12, the valid corner point in the first quadrant that satisfies the constraints and has the smallest X-coordinate is defined as endpoint $P_{1}$, whereas the valid corner point in the third quadrant that satisfies the constraints and has the largest Y-coordinate is defined as endpoint $P_{2}$, i.e.,

(18) $$P_{1}=\left\{p_{i}|p_{i}\in Q_{1},x_{i}=\min\left(x_{j}\right)\right\}$$

(19) $$P_{2}=\left\{p_{k}|p_{k}\in Q_{3},y_{k}=\max\left(y_{m}\right)\right\}$$

Here, $Q_{1}$ and $Q_{3}$ denote the sets of valid candidate points in the first and third quadrants, respectively. The purpose of introducing the extremum-based rule is to further select, under the constraint of the chamfer topology, the candidate corner point that lies closest to the geometric location of the target endpoint, thereby improving the uniqueness and stability of endpoint localization. Once the two target endpoints, $P_{1}$ and $P_{2}$, have been successfully determined, the pixel measurements of the corresponding chamfer features can be obtained and then converted into actual physical dimensions using the scale factors derived from the previous calibration procedure. In this way, the candidate point set produced by corner detection is decoupled from the subsequent local geometric-constraint-based screening process: the former is responsible for providing a sufficient set of candidates, whereas the latter uses contour structure and spatial constraints to achieve stable endpoint localization. While keeping the computational procedure concise, this method effectively suppresses the interference of background false corners, local contamination, and edge fluctuations in endpoint extraction, thereby providing a reliable basis for subsequent measurement of geometric parameters such as chamfer length, width, and height.

#### 2.4.3. Defect Determination Criteria

After chamfer endpoint localization and geometric parameter measurement have been completed, the chamfer quality status must be further evaluated. For samples with normal chamfers or only slight processing fluctuations, quality can be assessed according to the relationship between the measured dimensional parameters—such as chamfer length, width, and height—and the design tolerances. By contrast, under typical abnormal conditions such as edge chipping, corner breakage, missing edge grinding, and uneven grinding, the defects are often manifested as missing endpoint structures, abnormal spatial positions of the target endpoints, and imbalanced local geometric relationships. Accordingly, based on the endpoint localization and dimensional measurement results described above, this study establishes a set of defect discrimination criteria for transparent glass chamfers. These criteria integrate endpoint integrity, deviations in geometric parameters, and abnormal spatial distributions in the screening coordinate system to enable automatic identification of chamfer defects.

Based on the above considerations, three corner-detection-based criteria are proposed for diagnosing glass chamfer defects:

- Endpoint integrity criterion: After screening-coordinate processing, if fewer than two valid endpoints are detected, the sample is classified as having a structural defect. This is because a normal chamfer should contain at least two stably localizable feature endpoints in the corresponding inspection direction. Missing endpoints therefore usually indicate substantial local structural damage, such as corner loss, severe edge chipping, or missing edge grinding.
- Geometric consistency criterion: A sample is classified as having a morphological defect if any of the measured chamfer parameters—length, width, or height—exceeds the predefined tolerance threshold, or if there is a large discrepancy between the normal spacing of the transverse inner and outer boundary lines $X_{1}$ and $X_{2}$ and that of the longitudinal inner and outer boundary lines $Y_{1}$ and $Y_{2}$. In this context, abnormal chamfer width and height jointly indicate deviation of the chamfer angle, deviation in chamfer length reflects dimensional nonconformity, and a pronounced inconsistency between the two normal spacings corresponds to anomalies such as uneven grinding or local contour deformation.
- Quadrant-constraint anomaly criterion: For the standard chamfer orientation shown in Figure 12, if no candidate corner satisfying the constraints is detected in either the first or the third quadrant after the initial corner detection and coordinate-system screening, the sample is directly classified as defective. This rule is mainly used to identify spatial distribution anomalies caused by chamfer position offset, local contour distortion, or severe damage in the endpoint region. For chamfers with other rotated orientations, this criterion can be extended to the corresponding target quadrants according to the quadrant mapping relationship defined in Section 2.4.2.

**Figure 13:**
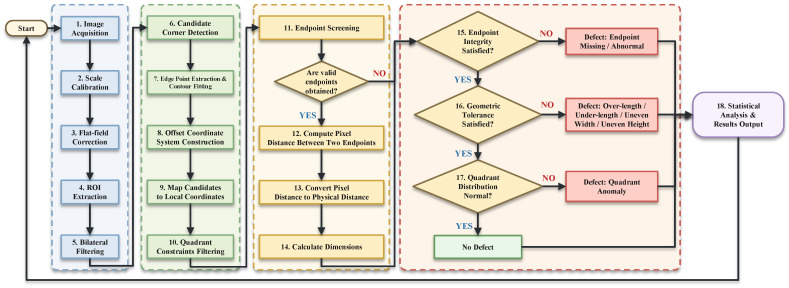
Overall workflow of the proposed corner-analysis-based glass chamfer inspection framework.

To improve the readability and reproducibility of the proposed method, the overall workflow of the corner-analysis-based glass chamfer inspection framework is summarized in Figure 13. The framework consists of five sequential stages: image acquisition and calibration, feature extraction and candidate generation, endpoint localization and dimensional measurement, quality assessment and defect determination, and statistical analysis. This workflow clarifies the processing order of the proposed method and shows how the endpoint localization results are further used for chamfer dimensional measurement, defect classification, and statistical evaluation.

To make the proposed inspection procedure easier to follow, the complete cor-ner-analysis-based glass chamfer inspection framework is summarized in Algorithm 1. The algorithm starts from image acquisition and scale calibration, followed by image correction, ROI extraction, and bilateral filtering. Candidate corner points are then generated and further screened through contour fitting, offset coordinate system construction, and quadrant constraints. Finally, the localized endpoints are used for dimensional measurement and defect determination, and the corresponding measurement errors, inspection time, and defect classification results are recorded for statistical analysis.

**Algorithm 1** Corner-analysis-based glass chamfer inspection framework
**Input:**
Raw line-scan image $I_{raw},$ flat-field image $I_{white},$ calibration parameters $k_{x}$ and $k_{y},$ predefined ROI, dimensional tolerance thresholds;
**Output:**
Chamfer dimensions (L,W,H), defect type, and inspection result;1.Acquire the raw chamfer image using the line-scan vision system;2.Calibrate the pixel-to-physical scale factors $k_{x}$ and $k_{y}$ in the x- and y-directions;3.Apply flat-field correction to obtain the corrected image $I_{corr};$4.Extract the predefined chamfer ROI from $I_{corr};$5.Apply bilateral filtering to suppress noise while preserving chamfer-edge features;6.Generate candidate corner points using the Shi–Tomasi detector to obtain candidate set;7.Extract high-confidence edge points and fit the inner and outer chamfer boundary lines;8.Construct the offset coordinate system based on the fitted boundary lines;9.Map the candidate corners into the local coordinate system;10.Apply quadrant constraints to remove non-target candidate points;11.Select the final chamfer endpoints using the extremum-based screening rules;12.Calculate the pixel distance between the two localized endpoints;13.Convert the pixel distance between the two localized endpoints into the actual physical distance;14.Calculate chamfer length (L), width (W), and height (H);15.Determine chamfer quality using endpoint integrity, geometric consistency, and quadrant-constraint anomaly criteria;16.Record measurement error, inspection time, and defect classification results for statistical evaluation.

## 3. Results and Discussion

### 3.1. Experimental Setup and Evaluation Metrics

#### 3.1.1. Dataset and Experimental Procedure

To validate the reliability and applicability of the proposed method in practical industrial scenarios, the experimental dataset was primarily composed of image samples collected at a production-line glass chamfer inspection station, including 524 normal chamfer samples and 117 defective chamfer samples. Given the limited number of defective samples available on the production line, and in order to cover typical abnormal conditions and improve the representativeness of the evaluation, the defective samples were drawn from two sources: one portion was collected directly from the production line, and the other was artificially constructed. The defect categories mainly included edge chipping, missing edge grinding, uneven grinding, and contamination by glass debris.

To evaluate the accuracy of chamfer dimensional measurement, key chamfer dimensions were manually measured using a high-precision caliper, and these measurements were used as the reference ground truth. For the defect detection task, sample acceptability and defect category were manually reviewed and confirmed by production-line quality inspectors, and these judgments were taken as the basis for evaluating defect classification results. The ground-truth data were matched one-to-one with the detection results for subsequent error analysis and detection-performance evaluation.

In the experimental procedure, all samples were validated using a unified processing pipeline. First, the input image was preprocessed. Candidate corners were then detected within the ROI, and a screening coordinate system was constructed in combination with geometric constraints to localize the chamfer endpoints. Once the endpoints had been determined, geometric parameters such as chamfer length, width, and height were measured. Finally, the inspection result was generated according to the defect discrimination criteria. The experiments were designed primarily to verify the stability and effectiveness of the overall method for both normal samples and multiple types of defective samples.

#### 3.1.2. Evaluation Metrics

To comprehensively evaluate the performance of the proposed method in dimen-sional measurement, defect discrimination, and online deployment, the following metrics were adopted.

(1) Dimensional measurement accuracy: Taking the caliper measurements as the reference ground truth, the errors in chamfer length (L), width (W), and height (H) were calcu-lated, and the mean absolute error (MAE) and maximum absolute error (MaxAE) were used as the primary accuracy metrics.
(2) Measurement success rate (MSR): This metric denotes the proportion of all samples for which endpoint localization was successfully completed and valid dimensional measurement results were obtained. If endpoint localization failed because of poor image quality, defect occlusion, or unsatisfied geometric constraints, the case was counted as a measurement failure.
(3) Defect detection performance: For defective samples, recall and false positive rate were calculated to evaluate the ability of the method to identify abnormal chamfers.
(4) Computational efficiency: The average processing time for a single chamfer ROI, in milliseconds, was recorded to assess the applicability of the method under production-line takt time requirements.

### 3.2. Dimensional Measurement Results and Accuracy Evaluation

To validate the effectiveness and stability of the proposed method for dimensional measurement of transparent glass chamfers, the normal chamfer samples were evenly divided into four groups, and the chamfer length, width, and height were measured separately. High-precision caliper measurements were used as the reference ground truth. For each sample, the algorithm first performed endpoint localization and then calculated the Chamfer dimensions on that basis. If valid dimensional results could not be obtained because endpoint localization failed or the geometric constraints were not satisfied, the case was counted as a measurement failure. The corresponding measurement results are presented in Table 7.

As shown in Table 7, the proposed method achieved stable dimensional measurement results for the four batches of normal chamfer samples. In the table, MAE denotes the mean absolute measurement error, MaxAE denotes the maximum absolute measurement error, and MSR denotes the measurement success rate. The average MAEs for chamfer length, width, and height were 0.102 mm, 0.080 mm, and 0.080 mm, respectively, resulting in an overall average MAE of 0.087 mm across the three dimensions. The corresponding average MaxAEs were 0.351 mm, 0.307 mm, and 0.300 mm, respectively, and the maximum error among all batches and dimensions was 0.390 mm. In addition, the measurement success rate (MSR) reached 100% for all four batches, and the average inspection time was 0.505 s per workpiece. These results indicate that the proposed method can stably localize chamfer endpoints and automatically measure key geometric parameters under actual production-line conditions.

In terms of error performance across different dimensional parameters, the measurement results for width and height were overall better than those for length. The average errors for both width and height were 0.080 mm, whereas the average error in the length direction was slightly higher at 0.102 mm. Based on the measurement procedure, this difference may be attributed to the larger endpoint spacing associated with the length parameter, which makes the measurement more sensitive to endpoint localization errors. By contrast, the measurement spans for width and height are smaller and are therefore less affected by local endpoint fluctuations and the accumulation of scale-conversion errors. Even so, the measurement error in the length direction remained within a relatively small range, indicating that the proposed endpoint screening and scale conversion methods are well suited to multidimensional measurement tasks.

**Figure 14:**
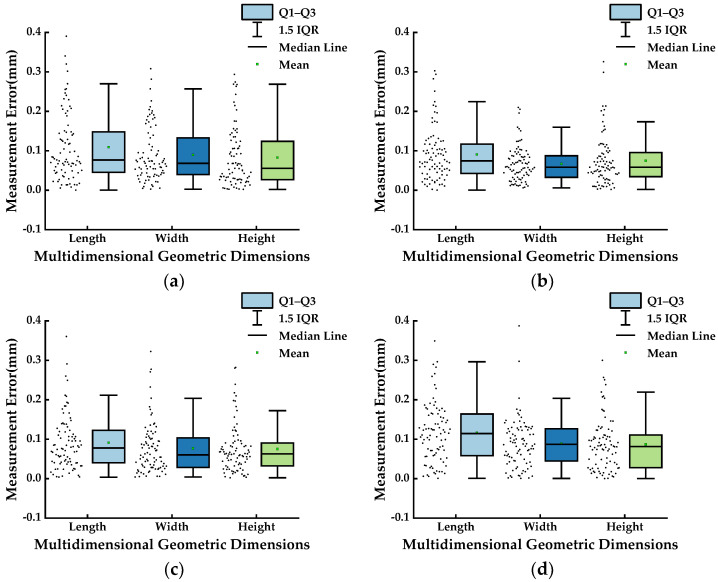
Boxplots of length, width, and height measurement errors for four batches of normal chamfer samples: (**a**) Batch 1, (**b**) Batch 2, (**c**) Batch 3, and (**d**) Batch 4.

To further illustrate the error distribution characteristics, Figure 14 presents boxplots of the measurement errors for the four groups of normal samples in the three dimensions of length, width, and height. As can be seen, the errors in each group are mainly concentrated, and the median values are generally below 0.15 mm, demonstrating good repeatability and stability of the proposed method under different batch conditions. The small number of outliers may be attributed to endpoint-neighborhood uncertainty caused by local reflections, weak edge contrast, or noise interference. However, these outliers are limited in number and do not materially affect the overall conclusion regarding measurement accuracy.

### 3.3. Defect Detection Results and Accuracy Evaluation

**Figure 15:**
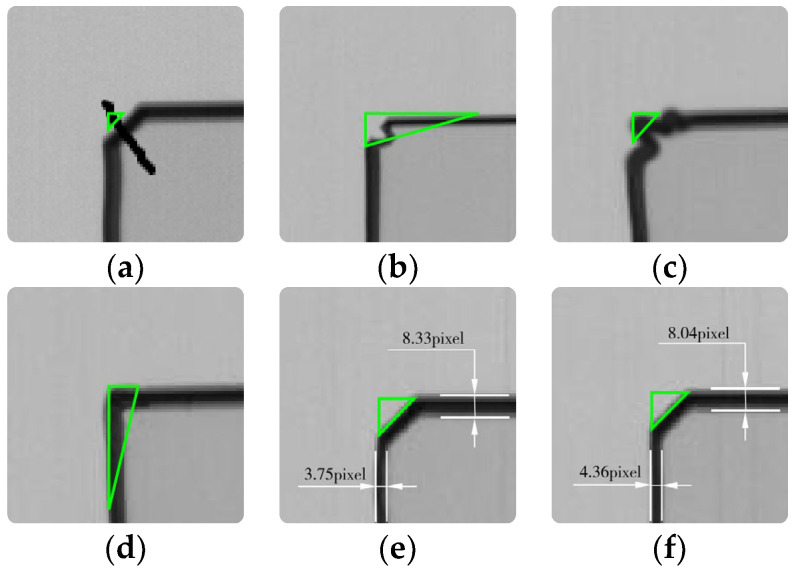
Representative examples of chamfer defect detection: (**a**) Debris contamination; (**b**) Edge chipping; (**c**) Edge chipping; (**d**) Missing edge grinding; (**e**) Non-uniform grinding; (**f**) Non-uniform grinding.

To validate the effectiveness of the proposed defect discrimination criteria, defect detection experiments were conducted on defective chamfer samples. After endpoint localization and feature extraction, each sample was evaluated for acceptability according to the established discrimination rules, and the correctness of the detection result was further confirmed through manual review. Figure 15 presents representative defective samples together with their detection results.

Given that the defect detection component mainly serves as a supplementary validation of quality assessment capability, and that the primary concern in production-line scenarios is the risk of missed detection, missed cases were taken as the main basis for analysis in this study. The experimental results show that the proposed method can effectively identify most chamfer defects characterized by missing endpoints, disrupted contour continuity, and abnormal geometric parameters. Among the 117 defective samples, 12 were missed and therefore not classified as defective, corresponding to an overall miss rate of 10.26%. The defective samples consisted of 63 real production-line defect samples and 54 artificially constructed defect samples. Among the real defect samples, 3 were missed, giving a miss rate of 4.76%. Among the artificially constructed defect samples, 9 were missed, giving a miss rate of 16.67%. The remaining 105 defective samples were correctly identified as containing chamfer defects. These results indicate that the proposed method can identify both real and artificially constructed defects, while the artificially constructed defects were more challenging in this study and showed a relatively higher risk of missed detection. Further analysis indicates that the missed detections occurred mainly in chipped-edge samples, most likely because contamination occlusion and grayscale disturbances in the acquired images made the defect features along the chamfer line less stable and therefore more difficult to detect. To further clarify the failure modes of the proposed method, the 12 missed cases were further categorized by defect type, and the corresponding image characteristics and causes of missed detection are summarized in Table 8.

The complete pipeline proposed in this study includes candidate corner generation, contour fitting, construction of an offset coordinate system, and quadrant-based endpoint screening for chamfer dimensional measurement and defect detection. Other methods, such as template matching and deep-learning-based defect detection, were not used as comparative pipelines in this study. Template matching mainly evaluates shape similarity and cannot directly provide quantitative Chamfer dimensional parameters, such as length, width, and height. In our preliminary tests, template matching was also sensitive to slight chamfer-edge disturbances and production-environment contamination, such as dust or debris, which could lead to false defect alarms. Deep-learning-based defect detection methods generally require a large number of well-annotated real production-line defect images for training and validation. However, in the current industrial scenario, real chamfer defect samples are relatively limited, and part of the defective dataset had to be artificially constructed to cover typical abnormal conditions. Therefore, compared with deep-learning-based methods, the proposed rule-based approach is more suitable for production-line scenarios where real defect samples are limited, defect annotation is costly, model interpretability is required, and online deployment efficiency is important. In future work, when a larger real-defect dataset becomes available, more comprehensive comparisons with data-driven methods will be conducted.

## 4. Conclusions

The aim of this study was to develop a non-contact, vision-based inspection method for transparent glass chamfers that can simultaneously achieve dimensional measurement and defect discrimination under online production-line conditions. The experimental results show that this aim was achieved. By combining image preprocessing, candidate corner generation, contour fitting, an offset screening coordinate system, and quadrant-based geometric constraints, the proposed method enabled stable chamfer endpoint localization and automatic measurement of chamfer length, width, and height.

In the dimensional measurement stage, tests on 524 normal chamfer samples showed average MAEs of 0.102 mm, 0.080 mm, and 0.080 mm for chamfer length, width, and height, respectively, with an overall mean absolute error of 0.087 mm. The measurement success rate reached 100%, and the average inspection time was 0.505 s per workpiece. In the defect discrimination stage, 105 of 117 defective samples were correctly identified, corresponding to a defect recall of 89.74% and a miss rate of 10.26%.

These results demonstrate that the proposed method provides a feasible and interpretable solution for automated online inspection of transparent glass chamfers. Its practical significance lies in integrating endpoint localization, dimensional measurement, and defect discrimination into a unified inspection framework, thereby reducing dependence on manual visual inspection and contact-based measurement. Future work will focus on collecting more real production-line defect samples, improving the recognition of subtle and weak-contrast defects, and enhancing the adaptability of the system to glass workpieces with different specifications and production conditions.

## Figures and Tables

**Table 1:** Calibration results of the scale coefficient in the x direction.

Measurement Point	Point 1	Point 2	Point 3	Point 4	Point 5	Point 6	Point 7	Point 8	Mean
Pixel length	2199.385	2198.697	2200.741	2200.155	2199.832	2200.233	2199.217	2200.652	2199.864
Scale coefficient	0.09548	0.09551	0.09542	0.09545	0.09546	0.09544	0.09549	0.09543	0.09546

**Table 2:** Calibration results of the scale coefficient in the y direction.

Measurement Point	Point 1	Point 2	Point 3	Point 4	Point 5	Point 6	Point 7	Point 8	Mean
Pixel length	3621.711	3622.343	3620.137	3622.482	3622.905	3622.261	3621.656	3620.734	3621.779
Scale coefficient	0.08283	0.08282	0.08287	0.08282	0.08281	0.08282	0.08284	0.08286	0.08283

**Table 3:** Verification results of *x*-direction scale calibration.

X-Direction Validation Group	1	2	3	4	5	6	7	8	Mean
Calculated length (mm)	209.880	209.975	210.008	209.756	209.649	210.232	209.868	209.739	209.888
Absolute measurement error (mm)	0.120	0.025	0.008	0.244	0.351	0.232	0.132	0.261	0.172

**Table 4:** Verification results of *y*-direction scale calibration.

Y-Direction Validation Group	1	2	3	4	5	6	7	8	Mean
Calculated length (mm)	299.673	300.184	299.904	300.251	299.582	300.205	299.857	299.714	299.921
Absolute measurement error (mm)	0.327	0.184	0.096	0.251	0.418	0.205	0.143	0.286	0.239

**Table 5:** Quantitative comparison of different filtering methods.

Method	PSNR	SSIM	EPI
Mean	28.3832	0.9361	0.8615
Median	35.9427	0.9771	0.9748
Gaussian	32.8347	0.9722	0.9574
Bilateral	36.8426	0.9868	0.9847

**Table 6:** Performance comparison of different candidate corner detectors.

Method	Valid Sample Size	N	Time (ms)	MLE (px)	MaxLE (px)	Recall	PR (%)	*p*-Value *
Harris	256	5.81 ± 1.77	5.554 ± 0.481	7.28 ± 1.62	11.63 ± 2.00	0.354 ± 0.241	28.52	<0.001
Shi–Tomasi	249	14.67 ± 2.11	6.222 ± 0.320	1.13 ± 0.12	1.42 ± 0.14	0.971 ± 0.083	88.76	—
FAST	255	6.34 ± 1.17	0.202 ± 0.019	2.37 ± 0.29	2.90 ± 0.32	0.925 ± 0.210	82.35	<0.05
ORB	263	34.28 ± 5.17	224.856 ± 21.243	1.79 ± 0.27	2.16 ± 0.32	0.884 ± 0.298	82.89	<0.05

* The *p*-value indicates the statistical significance of the MLE difference compared with Shi–Tomasi, as evaluated using Welch’s *t*-test. A value of *p* < 0.05 was considered statistically significant.

**Table 7:** Measurement results and accuracy evaluation for four batches of normal chamfer samples.

Batch	L MAE (mm)	L MaxAE (mm)	W MAE (mm)	W MaxAE (mm)	H MAE (mm)	H MaxAE (mm)	MSR (%)	Time (s)
1	0.109	0.390	0.090	0.308	0.083	0.294	100	0.497
2	0.091	0.303	0.067	0.210	0.075	0.326	100	0.526
3	0.091	0.360	0.077	0.322	0.075	0.282	100	0.508
4	0.116	0.349	0.089	0.387	0.087	0.300	100	0.488
Average	0.102	0.351	0.080	0.307	0.080	0.300	100	0.505

**Table 8:** Category Distribution and Cause Analysis of Missed Defect Detection Cases.

Defect Category	Number of Missed Detections	Main Reason for Missed Detection
Tiny protrusions along the chamfer line or slight edge chipping	6	The defect size was too small to significantly change the endpoint position, contour continuity, or geometric dimensions
Minor contamination or glass debris interference	2	The local grayscale disturbance was weak and could be regarded as normal texture or noise
Slight dimensional abnormality close to the threshold	4	The dimensional deviation was small and did not exceed the predefined defect judgment threshold
Total	12	/

## Data Availability

The original contributions presented in this study are included in the article. Further inquiries can be directed to the corresponding author.

## Abbreviations

The following abbreviations are used in this manuscript:

CNC	computer numerical control
DY-YOLO	dynamic You Only Look Once
EPI	edge preservation index
FAST	features from accelerated segment test
IQR	interquartile range
LED	light-emitting diode
MAE	mean absolute error
MaxAE	maximum absolute error
MSR	measurement success rate
ORB	oriented FAST and rotated BRIEF
PSNR	peak signal-to-noise ratio
ROI	region of interest
SDK	software development kit
SSIM	structural similarity index
TCP/IP	Transmission Control Protocol/Internet Protocol
YOLO	You Only Look Once
MLE	mean localization error
MaxLE	maximum localization error
PR	Perfect Rate
